# Asymmetrical Weakness Associated with Central Nervous System Involvement in a Patient with Guillain-Barrè Syndrome

**DOI:** 10.4137/ccrep.s3180

**Published:** 2009-09-03

**Authors:** Takao Kiriyama, Makito Hirano, Susumu Kusunoki, Daiji Morita, Minako Hirakawa, Yasuyo Tonomura, Takanori Kitauchi, Satoshi Ueno

**Affiliations:** 1 Department of Neurology, Nara Medical University School of Medicine, 840 Shijo-cho, Kashihara, Nara 634-8522, Japan.; 2 Department of Neurology, Kinki University School of Medicine. Email: hirano_makito@yahoo.co.jp

**Keywords:** Guillain-Barrè syndrome, GBS, symmetrical weakness, asymmetrical weakness, central nervous system, CNS

## Abstract

Guillain-Barrè syndrome (GBS) is usually associated with symmetrical weakness, and therefore asymmetrical weakness may confuse diagnosis. We report on a patient with GBS subsequent to *Campylobacter jejuni* enteritis who had asymmetrical weakness with CNS involvement. The patient tested positive for anti-ganglioside antibodies, including anti-GM1 IgM, anti-GD1b IgG, and anti-GT1a IgG. Patients with GBS can manifest asymmetrical signs and symptoms attributable to CNS involvement. Prompt, accurate diagnosis and treatment of post-*C. jejuni* GBS is especially important because its prognosis is relatively poor.

## Introduction

Patients with Guillain-Barré syndrome (GBS) typically present with symmetrical weakness and diminished deep tendon reflexes (DTR) due to peripheral neuropathy.^1^ Recent pathological and electrophysiological studies of GBS, however, have demonstrated involvement of the central nervous system (CNS), the clinical relevance of which remains uncertain.^2^,^3^ We here report on a patient with GBS who had asymmetrical weakness with CNS involvement.

## Patient

A 30-year-old man had diarrhea of 4-days’ duration. Two weeks after the onset of diarrhea, he was admitted to our hospital because of progressive weakness of the right arm and both legs. Neurologic examinations revealed asymmetrical weakness and deep tendon reflexes in all four limbs (Table 1). He had no extensor plantar response, sensory disturbance, respiratory failure, or cranial nerve involvement. A nerve conduction study (NCS) on the first hospital day showed only a slight decrease in the compound muscle action potential (CMAP) of the right (rt) median nerve (8 mV [normal ≥ 9.9 mV]). By contrast, studies of motor evoked potential (MEP) bilaterally revealed non-detectable potentials at the abductor pollicis brevis in response to cortical stimulation, but normal latency in response to cervical-root stimulation, indicating CNS involvement. This notion may be further supported by normal F-wave frequencies/latencies in all tested nerves, likely excluding proximal root involvement.^4^ A-waves were not detected. Cerebrospinal fluid (CSF) contained normal cell count (1 cell/μl) and protein concentration (37 mg/dl) on admission, but an increased protein concentration (136 mg/dl) with a normal cell count (5 cells/μl) 3 weeks after onset, a finding called albuminocytological dissociation that is typically found in GBS. Multiple sclerosis and other inflammatory diseases with CNS involvement were unlikely, because of no abnormalities on plain or contrast MRI of the entire spinal cord or brain and because of negative oligo clonal IgG bands, normal IgG index (0.36), and normal myelin basic protein in CSF. In addition, he had no ophthalmological abnormalities. Serum contained no autoantibodies against Sm, RNP, SS-B, Jo-1, Scl-70, ss-DNA, ds-DNA, neutrophil cytoplasm (PR3 and MPO), acetylcholine receptor, or thyroglobulin, or rheumatoid factor. However, the patient was seropositive for anti-*Campylobacter jejuni* antibodies. Measurement of anti-ganglioside antibodies using GM1, GM2, GM3, GD1a, GD1b, GD3, GT1b, GQ1b, GA1, and Gal-C as antigens according to the previous method^5^ revealed positive anti-GM1 IgM (OD = 0.275), anti-GD1b IgG (0.265), and anti-GT1a IgG (0.207) antibodies (normal <0.100 for both IgM and IgG). These anti-ganglioside antibodies became negative 8 months after onset. Follow-up NCS for the median, ulnar, and tibial nerves showed that CMAP reached nadirs 2–3 weeks after onset (rt/left [lt] median nerve, 5.07/1.64 mV [normal ≥9.9 mV]; rt/lt ulnar nerve, 7.74/2.62 mV [normal ≥9.5 mV], and rt/lt tibial nerve 3.33/3.98 mV [normal ≥9.3 mV]). However, data of F-wave frequencies/latencies, motor conduction velocities, distal latencies, sensory nerve conduction velocities, sensory nerve action potentials including sural nerves were almost normal during the entire disease course.

The patient responded to intravenous immunoglobulin (IVIG) therapy, started on the first hospital day. Nine months later, the patient still had mild weakness of the left hand. The central motor conduction time (CMCT) on a MEP study was calculated as described previously (CMCT = MEP latency—[F wave latency + M wave latency −1]/2).^3^ The CMCT of the abductor pollicis brevis was delayed on the left (10.8 msec [normal 5.3–8.2 msec]), but normal on the right (5.7 msec). The left hand weakness and delayed CMCT resolved 1 year after disease onset. No recurrence was noted for more than 6 years.

## Discussion

We have described a patient who had GBS with CNS involvement as confirmed electrophysiologically. The diagnosis of GBS may have been supported by a recent history of diarrhea, which was later associated with positive anti-*C. jejuni* and anti-ganglioside antibodies. Because GBS is characterized by symmetrical weakness,^6^ the marked asymmetry in our patient might have been at least partly ascribed to CNS involvement. In particular, prolonged weakness of the left hand closely correlated with the delayed CMCT. By contrast, peripheral nerve involvement was negligible initially, but mild subsequently. Despite such an atypical pattern of clinical and electrophysiological signs and symptoms, IVIG therapy, begun on the day of admission, apparently contributed to the functional recovery of our patient.

Previous reports have described patients who had anti-ganglioside-antibody-associated neuropathy with hyperreflexia and mildly delayed CMCT.^3^ In contrast, our patient had normal to decreased DTR. Although the reason for the difference in DTR between the present and previous patients remains unclear, distinct combinations of central and peripheral involvement might be responsible. The peripheral involvement in our patient may have partly masked the central involvement typically associated with hyperreflexia. Alternatively, the observed hyporeflexia may have also been a manifestation of severe CNS involvement, as often seen in acute myelitis.^7^ Clearer definition of the mechanisms involved requires further studies.

One would speculate that the absence of hyperreflexia, extensor plantar response, or MRI abnormalities excludes CNS involvement. However, in multiple sclerosis, a disease predominantly or almost exclusively involving CNS, a MEP study may more sensitively detect CNS involvement than the clinical examinations, since only 58% patients with MEP abnormalities were positive for extensor plantar response and 72% were positive for hyperreflexia.^8^ MRI is one of the most sensitive methods to detect CNS abnormalities, such as demyelination and edema often found in multiple sclerosis. However, normal MRI findings cannot exclude CNS involvement since subacute combined degeneration of the spinal cord, a typical form of myelopathy, showed normal MRI findings in more than 40% of patients.^9^ In addition, a certain type of myelitis lacked MRI abnormalities.^10^ A recent review demonstrates that MEP studies are occasionally superior to radiological tests for detecting myelopathy.^11^ Thus, the MEP study performed here may have sensitively detected CNS involvement in our patient.

Pathological examination of the CNS was not performed in our patient, who recovered functionally. Previous autopsy studies of patients with GBS have provided evidence of CNS involvement, including central chromatolysis of motor neurons, mononuclear cell filtration, and microglial activation in the lateral column of the spinal cord.^2^ Given the reversible CNS involvement in our patient, the mononuclear cell filtration and microglial activation in the lateral column might represent a primary pathological change.

Finally, it should be noted that patients with GBS can manifest asymmetrical signs and symptoms, possibly associated with CNS involvement. Prompt, accurate diagnosis and treatment of post-*C. jejuni* GBS is mandatory, given the relatively poor prognosis.^12^

## Figures and Tables

**Table 1:** Clinical signs and symptoms on admission.

	Rt	Lt
Grip strength	40 kg	25 kg
*MMT*^a^		
Deltoid	1	5
Biceps brachii	2	5
Triceps	5	5
Wrist extensor	5	4
Wrist flexor	4	4
Abductor pollicis brevis	4	4
Iliopsoas	3	3
Quadriceps	5	5
Hamstrings	5	5
Tibialis anterior	5	3
Gastrocnemius muscles	3	5
*DTR*^b^		
Biceps brachii	↓	N
Triceps	↓	N
Brachioradialis	N	↓
Patella	↓	N
Achilles’ tendons	↓	N

a manual muscle test: 0 = no muscular contraction, 5 = full strength,

b deep tendon reflex: ↓, decreased; N, normal.
